# Use of Complementary and Alternative Medicine Among Patients With Arthritis

**Published:** 2009-03-15

**Authors:** Leigh F. Callahan, Elizabeth K. Wiley-Exley, Thelma J Mielenz, Changfu Xiao, Shannon S. Currey, Betsy L. Sleath, Philip D. Sloane, Robert F. DeVellis, Teresa J. Brady, Joseph Sniezek

**Affiliations:** Thurston Arthritis Research Center; University of North Carolina at Chapel Hill, Chapel Hill, North Carolina; University of North Carolina at Chapel Hill, Chapel Hill, North Carolina; University of North Carolina at Chapel Hill, Chapel Hill, North Carolina; University of North Carolina at Chapel Hill, Chapel Hill, North Carolina; University of North Carolina at Chapel Hill, Chapel Hill, North Carolina; University of North Carolina at Chapel Hill, Chapel Hill, North Carolina; University of North Carolina at Chapel Hill, Chapel Hill, North Carolina; Centers for Disease Control and Prevention, Atlanta, Georgia; Centers for Disease Control and Prevention, Atlanta, Georgia

## Abstract

**Introduction:**

Previous studies suggest that people with arthritis have high rates of using complementary and alternative medicine (CAM) approaches for managing their arthritis, in addition to conventional treatments such as prescription medications. However, little is known about the use of CAM by diagnosis, or which forms of CAM are most frequently used by people with arthritis. This study was designed to provide detailed information about use of CAM for symptoms associated with arthritis in patients followed in primary care and specialty clinics in North Carolina.

**Methods:**

Using a cross-sectional design, we drew our sample from primary care (n = 1,077) and specialist (n = 1,063) physician offices. Summary statistics were used to calculate differences within and between diagnostic groups, practice settings, and other characteristics. Logistic regression models clustered at the site level were used to determine the effect of patient characteristics on ever and current use of 9 CAM categories and an overall category of "any use."

**Results:**

Most of the participants followed by specialists (90.5%) and a slightly smaller percentage of those in the primary care sample (82.8%) had tried at least 1 complementary therapy for arthritis symptoms. Participants with fibromyalgia used complementary therapies more often than those with rheumatoid arthritis, osteoarthritis, or chronic joint symptoms. More than 50% of patients in both samples used over-the-counter topical pain relievers, more than 25% used meditation or drew on religious or spiritual beliefs, and more than 19% used a chiropractor. Women and participants with higher levels of education were more likely to report current use of alternative therapies.

**Conclusion:**

Most arthritis patients in both primary care and specialty settings have used CAM for their arthritis symptoms. Health care providers (especially musculoskeletal specialists) should discuss these therapies with all arthritis patients.

## Introduction

More than 1 in 5 US adults (46.4 million people) had doctor-diagnosed arthritis in 2003, and that number will grow to an estimated 67 million by 2030 (1). Arthritis is a common cause of disability in the United States, and the costs are substantial, estimated to be approximately 1.2% of the US gross domestic product (2).

Proper management of arthritis can reduce pain, functional limitations, and related problems (3). Treatment and management of arthritis can include medication, physical or occupational therapy, patient education, weight loss, and surgery. Increasingly, complementary and alternative medicine (CAM) therapies are also being used. These therapies are a group of practices or products that are not currently used in the practice of conventional medicine. Estimates of CAM ever use among adults with arthritis range from 59% (4) to 90% (5,6).

Many studies have looked at CAM use for arthritis (5-14), but we were particularly interested in the frequencies of use for multiple types of CAM by diagnostic category (especially within larger categories) and other characteristics, for which few data are available. Most studies provide only the averages for each category, which masks these differentiations. Herman et al (5) found that 23.7% of people with arthritis in a sample from New Mexico used glucosamine, but only 1.2% used gamma linolenic acid. Katz and Lee (6) found that, although 42.4% of people with arthritis across the United States used mind-body interventions (such as prayer, spiritual healing, and biofeedback), only 3.7% used some form of relaxation therapy, guided imagery, or positive imagery. More information from populations from different parts of the country would provide an adjunct to these findings. We also explored the use of CAM by people with arthritis seeing different types of health care providers.

Our main objective was to provide detailed information about ever and current use of methods of CAM for symptoms associated with osteoarthritis (OA), rheumatoid arthritis (RA), fibromyalgia (FM), and chronic joint symptoms (CJS) by demographic and disease status characteristics in a sample of 2,140 people in North Carolina. Our secondary objective was to separate and describe these findings by type of practice setting, primary care or specialty.

## Methods

### Recruitment

Samples were drawn from 2 populations based on a study protocol approved by the University of North Carolina institutional review board: a family medicine research network and a musculoskeletal database.

**Family Medicine Research Network**

Data from the primary care setting were gathered via the North Carolina Family Medicine Research Network (NC-FM-RN), described in detail by Sloane et al (15). During 2001, research assistants approached all adult patients in a representative sample of 16 family practice sites during a 4-week period. Each consenting adult patient was administered a 4-page self-report survey with questions on demographics, self-reported chronic conditions, health habits (eg, smoking and physical activity), and self-rated health.

The racial/ethnic composition of the 5,575 patients who agreed to participate reflected that of the state's adult population in terms of African Americans, Hispanics, and adults aged 65 or older. Patients who self-reported RA, OA, FM, or CJS were asked to complete the survey (n = 2,026).

**Musculoskeletal database**

The musculoskeletal database was established in the mid-1990s as part of an ongoing, longitudinal project measuring arthritis outcomes. During an outpatient visit, patients seen in the rheumatology or orthopedic clinics at the University of North Carolina Hospitals or 13 selected private rheumatology practices in North Carolina were asked to participate. Patients who agreed to participate completed a consent form and baseline self-report questionnaire on demographic and health-related characteristics; diagnosis and date of disease onset were provided by the patient's physician. Patients with RA, OA, or FM who completed this process and agreed to further contact were mailed the survey (n = 2,075).

### Survey

Two survey booklets were mailed to 4,101 people. The first asked about health, health beliefs, and use of health care. The second asked about use of CAM. After 3 weeks, nonrespondents were sent a second set of survey booklets, and then were contacted by telephone if neither mail survey elicited responses. A total of 2,140 patients responded to the survey (52.2%); 1,077 were from the NC-FM-RN, and 1,063 were from the musculoskeletal database.

### Measures

**Characteristics**

Demographic characteristics included age, sex, race/ethnicity, education level, location of practice (urban or rural), and marital status. Because of the small number of responses in the categories other than African American or white, responses were categorized into white, African American, or other. Education was based on self-reported number of years, and marital status was dichotomized into currently married or not.

**Disease**

Disease information included self-reported RA, OA, FM, or CJS for the NC-FM-RN sample. The category of CJS was used if patients reported having had symptoms of pain, aching, or stiffness in or around joints during the last 30 days and did not self-report having RA, OA, or FM. For the specialist sample, the primary diagnosis (RA, OA, or FM) was provided by the specialist. Each participant in the 2 samples was then assigned a primary diagnosis of RA, OA, or FM (with CJS also assigned in the NC-FM-RN dataset only). Consistent with previous research (5), we classified participants who had more than 1 type of arthritis in the following order of priority: 1) RA, 2) FM, and 3) OA. In the NC-FM-RN dataset, 192 patients (18%) were classified with RA, 400 (37%) with OA, 81 (8%) with FM, and 404 (38%) with CJS; in the musculoskeletal database, 489 patients were classified with RA (46%), 300 (28%) with OA, and 274 (26%) with FM.

**Health Assessment Questionnaire (HAQ)**

The HAQ disability scale (16,17) is a reliable and valid instrument that rates difficulty with 20 activities of daily living ranging from 0 (without any difficulty) to 3 (unable to do). We calculated an unweighted mean of these scores.

**Sleep**

Four questions focused on sleep ("Do you have trouble falling asleep?," "Do you wake up several times per night?," "Do you have trouble staying asleep?," and "Do you wake up after your usual amount of sleep feeling tired and worn out?") (18). The scores could range from 0 (no problems) to 5 (the most problems). We calculated an unweighted mean of these scores.

**Pain and fatigue**

Visual analog scales (VASs) were used to measure pain and fatigue (19). For example, the amount of pain experienced during the past week was assessed by using a 100 mm VAS anchored with "no pain" (0 mm) and "pain as bad as it could be" (100 mm).

**Rheumatology Attitudes Index (RAI)**

The 5-item helplessness subscale of the RAI (20) was used to measure perceived helplessness (ie, the degree to which one believes the condition of interest is controlling one's life). Five questions were scored on a scale from 1 to 5, with 5 being the most helpless, and an unweighted mean of these scores was calculated.

**CAM**

Participants were asked about 9 categories of CAM use: alternative providers, special diets, vitamins and minerals, supplements, ointments or topical rubs, body treatments (eg, copper bracelets and magnets), movement (eg, yoga), spiritual (eg, prayer), and mind-body therapies (eg, visualization). In the regression models and when totals are reported for the category of vitamins and minerals, the following were excluded because they are often prescribed or strongly suggested by physicians for people with musculoskeletal disorders: multivitamins, calcium, folic acid, and vitamin D. The specific percentage for each of these categories, however, is provided. A final (10th) category of "any use" was computed, which was coded as yes if the participants were using any of the 9 categories of CAM. Participants were asked whether they 1) have "ever used [therapy] for your arthritis or joint symptoms," 2) "currently use [therapy] for your arthritis or joint symptoms," and 3) "plan to continue to use [therapy] for your arthritis or joint symptoms."

### Statistical analysis

Summary statistics were calculated; proportions are given for categorical variables, and means with the standard deviation are given for continuous variables. We used χ^2 ^and linear regression with dummy variables to determine significant differences within and between diagnostic groups, practice settings, and other demographic characteristics. Logistic regression models clustered at the site level were used to determine the effect of patient characteristics on current use of the 9 CAM categories and "any use" by using Stata software version 9.0 (StataCorp LP, College Station, Texas). Models were adjusted for age, sex, race, education, marital status, HAQ score, RAI score, pain VAS, fatigue VAS, and location of practice.

## Results

### Demographics

Higher proportions of participants were women and were white in both samples (Table 1). Approximately half of the participants had more than a high school education. Almost half of patients in the primary care sample received care from rural practices, and all patients in the specialist sample received care from urban practices. The mean age in the specialist sample was slightly higher (59.8 years vs 54.0 years).

### Types of CAM used

More than 80% of both samples had used some form of CAM for arthritis symptoms during the course of their disease (data not shown). Ointments or topical rubs were the most commonly used CAM (Table 2). More than 60% of both groups had ever used rubs. Spiritual methods were the second most commonly used CAM category; approximately 40% to 49% of participants had ever used them. Alternative providers, vitamins and minerals, other supplements, movement, and mind-body therapies were ever used by 22% to 40% of the groups. Special diets, on the other hand, were the least commonly used (7% to 16% of both groups ever used special diets).

Although rubs were the most common ever-used CAM category, the rates of current use were much lower (approximately half). The same was true for alternative providers and body treatments (eg, magnets). However, rates of ever and current use were similar for special diets, spiritual methods, and mind-body therapies.

Of the most commonly used specific types of CAM (Table 3), more than 50% of both samples used Bengay, Icy Hot, or similar ointments or rubs; more than 25% used meditation or drew upon religious or spiritual beliefs; and more than 20% had seen a chiropractor or used calcium supplementation.

In the musculoskeletal database, 90.5% had used at least 1 CAM therapy for their arthritis symptoms during their disease course, and 75.9% still used at least 1 CAM therapy at the time of the survey (data not shown). For the NC-FM-RN sample, a smaller percentage (82.8%) had ever tried at least 1 CAM therapy, and 70.2% were still using at least 1 CAM therapy at the time of the interview (data not shown). Methods used by 20% of patients in both settings included chiropractors; calcium; Bengay, Icy Hot, and similar ointments or rubs; spiritual beliefs; and meditation.

Participants with FM used CAM therapies more often than did those with RA, OA, or CJS (Table 2). Of the specific categories of CAM use (Table 3) that showed significant differences (*P* < .05) in use by disease category, patients with FM used most CAM therapies significantly more often than those with other types of arthritis.

For both sets of participants with OA, meditation was also commonly used (35.8% for primary, 34.7% for specialty), as were drawing on spiritual beliefs and meditation for participants with FM in the NC-FM-RN setting (55.6% for both CAM therapies).

### Characteristics of current CAM users

In logistic regression models adjusted for age, sex, race, education, marital status, disability, pain, fatigue, and practice location, only sex was significantly associated with current use of any CAM in all 9 categories (data not shown). Most CAM therapy categories were significantly associated with at least 2 patient characteristics; for example, sex, race, and education were associated with the current use of supplements. However, sex was the only characteristic significantly associated with current use of special diets.

Female sex was positively associated with most categories of CAM use, while higher levels of education were positively associated with 5 categories of CAM use and negatively associated with current use of ointments or topical rubs. Of the other characteristics included in the adjusted analyses, the categories of African Americans, whites, and other race were positively associated with 3 categories of current CAM use: supplements, ointments and topical rubs, and spiritual. Rural location of the practice was negatively associated with current use of 2 categories: CAM providers and body treatments (eg, magnets). Disability, measured by the HAQ, was positively associated with spiritual and mind-body therapy categories. Helplessness, measured by the RAI, was positively associated with body treatments.

## Discussion

In this survey of 2,140 people with arthritis in North Carolina, most had used some form of CAM for their arthritis symptoms. This finding is close to other estimates (5,6) of 90.2% and 80% of ever use or use within the past month, although it is much higher than findings of 34% to 68% from many earlier studies (7,8,12,14,21).

Some of the differences between our study and earlier studies that reported much lower levels of ever use of CAM may be attributable to our inclusion of prayer. In our study, 13.7% of the family practice group and 17.4% of the specialty group prayed about their arthritis. Almost half (40.6%) of the sample with OA of the knee from Katz and Lee (6) used prayer. The numbers reported by Cronan et al (22) also included prayer as a form of CAM, and their findings of ever use were similar.

However, this inclusion does not seem to explain all of the difference, because Herman et al (5) did not include prayer but still had similar findings. They attribute their higher percentage of use to differing definitions of CAM, noting that they surveyed for a broader array of mind-body therapies, energy therapies, and CAM movement therapies than most other studies. They also suggested that the differences between their study and earlier studies were attributable to geographic location, noting that CAM use is often higher in the Western regions of the United States, where their study took place.

A larger proportion of participants from the specialty setting had used CAM than had participants from the family practice setting. This finding is not surprising because patients seeing specialists have more severe disease (23) and are probably in need of greater pain relief. Our findings corroborate a study by Breuer et al (11) that noted significantly more CAM use by patients with FM and a study by Herman et al (5) that reported a higher number of CAM therapies used by patients with FM and RA than those with OA. The higher use of CAM therapies by participants with FM compared with participants who have other forms of arthritis is also not surprising. Few good pharmacologic treatments are available for FM, and people with FM are often encouraged to participate in exercise regimens and meditation, which could account for some of the higher levels of use (24-26). In addition, people with FM experience a wide variety of symptoms, such as nonrestorative sleep, mood disturbance, irritable bowel syndrome, headache, and paraesthesias (25,27). These symptoms may catalyze the use of a broader range of therapies.

Participants in our survey tried a variety of therapies, and although many tried rubs, alternative providers, and body treatments, they often were not currently using those methods. Ever and current use of special diets, spiritual methods, and mind-body therapies, on the other hand, were similar. This could suggest that people with arthritis are more satisfied with dietary, spiritual, and mind-body methods. More research in this area might explore what it is about these methods that promotes continued use.

Several limitations should be noted when interpreting these results. Most prominently, the CAM questions in our survey asked whether respondents used CAM for arthritis or joint symptoms. Participants conceivably could have misread the question as asking whether they had ever used CAM for any reason. This issue has arisen in previous research (5), and validation of this aspect of the questionnaire is needed. Similarly, the self-reported nature of the diagnoses for participants in the family practice group is potentially problematic. Self-reported data for arthritis reportedly have moderate sensitivity (71%) and specificity (70%), but few studies address the issue (28).

This study also is limited in its ability to determine the use of CAM among races/ethnicities other than African American and white. Other studies have looked more closely at this issue (5,6). Although our study's ethnic composition at enrollment paralleled that of the state's adult population, oversampling of some races/ethnicities, such as Asians and Hispanics, would have enabled us to say more about these populations. In addition, these findings are based on a cross-sectional survey. The findings from previous research show that people frequently change their patterns of CAM use (7). For this and other reasons, we have focused on both ever and current use in this article.

Because almost every participant in our study used CAM at some point for his or her arthritis symptoms, it may be useful for practitioners to invite discussion of what therapies patients might be using for their symptoms and to assist them in evaluating risks.

## Figures and Tables

**Table 1:** Patient Characteristics by Diagnostic Group and Practice Setting, Among a Sample of Patients From North Carolina With Musculoskeletal Disorders, 2001

Characteristic	Rheumatoid Arthritis	Osteoarthritis	Fibromyalgia	Chronic Joint Symptoms	*P* a	All	*P* b
**No. of patients**							
Primary	192	400	81	404	NC	1,077	NC
Specialty	489	300	274	NR	NC	1,063	
**Female, %**							
Primary	77.3	75.4	97.5	74.4	<.001	77.0	.03
Specialty	73.4	79.3	96.0	NR	<.001	80.9	
**Race, %**							
**White**							
Primary	69.5	81.5	91.4	72.9	NC	76.9	NC
Specialty	83.2	81.8	88.2	NR	NC	84.1	
**African American**							
Primary	27.3	15.2	4.9	23.6	NC	19.7	NC
Specialty	14.3	15.4	9.5	NR	NC	13.3	
**Other**							
Primary	3.2	3.3	3.7	3.5	<.001	3.4	<.001
Specialty	2.5	2.9	2.3	NR	.29	2.5	
**Education, %**							
**Less than high school graduate**							
Primary	35.5	24.6	10.0	16.7	NC	22.5	NC
Specialty	18.9	19.7	9.7	NR	NC	16.7	
**High school graduate**							
Primary	29.0	29.2	21.3	32.2	NC	29.7	NC
Specialty	34.5	26.3	34.3	NR	NC	32.2	
**More than high school graduate**							
Primary	35.5	46.2	68.8	51.1	<.001	47.9	.004
Specialty	46.6	54.0	56.0	NR	.001	51.1	
**Rural location of practice^c^, %**							
Primary	56.8	49.3	49.4	49.0	.30	50.5	NC
**Married, %**							
Primary	52.4	59.5	66.7	59.5	.15	58.8	<.001
Specialty	68.3	65.8	72.9	NR	.18	68.8	
**Mean age, y (SD)**							
Primary	57.7 (13.9)	59.7 (13.6)	51.3 (11.8)	47.3 (13.9)	<.001	54.0 (14.7)	<.001
Specialty	60.0 (12.2)	65.2 (12.3)	53.5 (11.5)	NR	<.001	59.8 (12.8)	

Abbreviations: NC, not calculated; NR, not reported.

a *P* value across diagnoses within the North Carolina Family Medicine Research Network (NC-FM-RN) or specialist for χ^2^ or linear regression with dummy variables as appropriate.

b *P* value between NC-FM-RN and specialist for χ^2^ or 2-sample *t* tests as appropriate.

c All specialty practices were urban.

**Table 2:** Ever and Current Use of Categories of CAM Therapies, by Diagnostic Group and Practice Setting Among a Sample of Patients From North Carolina With Musculoskeletal Disorders, 2001

Type of CAM, %	Rheumatoid Arthritis	Osteoarthritis	Fibromyalgia	Chronic Joint Symptoms	*P* a	All	*P* b
**Alternative providers^c^**							
**Ever**							
Primary	31.3	34.3	58.0	30.0	<.001	33.9	.50
Specialty	23.7	35.7	55.5	NR	<.001	35.3	
**Current**							
Primary	12.5	13.5	34.6	12.9	<.001	14.7	.23
Specialty	11.7	14.3	27.7	NR	<.001	16.6	
**Special diets^d^**							
**Ever**							
Primary	13.5	12.8	24.7	5.7	<.001	11.1	.002
Specialty	14.5	12.7	21.2	NR	.01	15.7	
**Current**							
Primary	8.3	9.0	17.3	3.5	<.001	7.4	.01
Specialty	9.2	9.0	14.2	NR	.06	10.4	
**Vitamins and minerals^e^**							
**Ever**							
Primary	30.7	30.5	50.6	16.8	<.001	26.9	<.001
Specialty	36.8	33.0	46.7	NR	.002	38.3	
**Current**							
Primary	22.9	22.8	37.0	13.9	<.001	20.5	<.001
Specialty	28.8	26.0	35.4	NR	.04	29.7	
**Supplements^f^**							
**Ever**							
Primary	28.7	31.0	33.3	16.6	<.001	25.4	<.001
Specialty	33.1	42.7	50.0	NR	<.001	40.2	
**Current**							
Primary	18.8	22.0	21.0	11.4	.001	17.4	.03
Specialty	16.0	25.3	25.6	NR	.001	21.1	
**Ointments or topical rubs**							
**Ever**							
Primary	64.1	61.8	64.2	56.4	.21	60.4	.84
Specialty	55.8	63.3	66.8	NR	.007	60.8	
**Current**							
Primary	37.5	38.3	39.5	34.6	.69	36.9	<.001
Specialty	22.7	32.3	37.2	NR	<.001	29.2	
**Body treatments^g^**							
**Ever**							
Primary	29.2	27.3	37.0	18.3	<.001	25.0	<.001
Specialty	35.4	33.3	44.5	NR	.01	37.2	
**Current**							
Primary	11.5	10.8	16.1	7.2	.06	9.9	.07
Specialty	9.2	11.7	19.0	NR	<.001	12.4	
**Movement^h^**							
**Ever**							
Primary	20.8	22.3	35.8	21.5	.03	22.8	<.001
Specialty	24.5	32.0	43.1	NR	<.001	31.4	
**Current**							
Primary	14.1	14.5	23.5	15.6	.21	15.5	<.001
Specialty	18.0	21.7	28.5	NR	.003	21.7	
**Spiritual^i^**							
**Ever**							
Primary	50.5	39.0	64.2	31.4	<.001	40.1	<.001
Specialty	50.5	39.0	58.0	NR	<.001	49.2	
**Current**							
Primary	42.7	36.8	59.3	28.5	<.001	36.4	<.001
Specialty	45.4	35.0	55.8	NR	<.001	45.2	
**Mind-body therapies^j^**							
**Ever**							
Primary	24.5	27.5	56.8	23.5	<.001	27.7	<.001
Specialty	31.1	30.0	52.2	NR	<.001	36.2	
**Current**							
Primary	20.3	23.5	56.8	20.8	<.001	24.4	<.001
Specialty	26.8	25.3	47.5	NR	<.001	31.7	

Abbreviation: SD, standard deviation.

a *P* value across diagnoses within North Carolina Family Medicine Research Network (NC-FM-RN) or specialist for χ^2^.

b *P* value between NC-FM-RN and specialist for χ^2^.

c Includes health providers and therapists who are not medical doctors.

d For example, arthritis diet or vegan diet.

e Excluding calcium, folic acid, vitamin D, and multivitamins.

f For example, aloe vera or fish oil.

g For example, copper bracelets or magnets.

h Physical activities.

i For example, prayer or attending religious services.

j For example, visualization or relaxation.

**Table 3:** Ever Use of Specific CAM Modalities by Diagnostic Group and Practice Setting Among a Sample of Patients From North Carolina With Musculoskeletal Disorders, 2001

Type of CAM, %	Rheumatoid Arthritis	Osteoarthritis	Fibromyalgia	Chronic Joint Symptoms	*P* a	All
**Alternative providers**						
**Acupuncturist**						
Primary	4.7	3.0	17.3	3.2	<.001	4.5
Specialty	4.3	8.3	20.8	NR	<.001	9.7
**Ayurvedic doctor**						
Primary	0.5	0	0	0	.25	0.1
Specialty	0.2	0.3	0.4	NR	>.99	0.3
**Chiropractic doctor**						
Primary	22.4	20.0	42.0	20.8	<.001	22.4
Specialty	11.7	21.0	32.9	NR	<.001	19.8
**Curandero/curandera^b^**						
Primary	1.0	0.3	0	0.3	.51	0.4
Specialty	0	0	0	NR	>.99	0
**Doctor of Oriental medicine**						
Primary	0.5	1.0	6.2	0.7	.007	1.2
Specialty	0.6	0.7	4.4	NR	<.001	1.6
**Herbalist, yerbero**						
Primary	1.0	1.0	3.7	1.0	.24	1.2
Specialty	1.4	0	3.7	NR	.001	1.6
**Homeopathic practitioner**						
Primary	0.5	1.0	2.5	1.0	.56	1.0
Specialty	1.0	0.3	3.7	NR	.004	1.5
**Hypnotist**						
Primary	0.5	1.0	1.2	0.3	.33	0.7
Specialty	0.2	0.3	1.8	NR	.03	0.7
**Iridologist**						
Primary	1.0	0.3	0	0.7	.53	0.6
Specialty	0.6	0.7	1.1	NR	.82	0.8
**Massage therapist, sobador**						
Primary	8.3	8.8	30.9	8.2	<.001	10.1
Specialty	7.6	11.0	32.9	NR	<.001	15.1
**Myofascial therapist**						
Primary	0	0.8	4.9	0.3	.002	0.7
Specialty	1.0	0.7	5.1	NR	<.001	2.0
**Naturopathic doctor**						
Primary	1.6	0.3	1.2	0.5	.20	0.7
Specialty	1.0	0.3	2.2	NR	.12	1.1
**Osteopathic doctor**						
Primary	3.7	5.0	7.4	1.7	.03	3.7
Specialty	4.1	6.3	5.1	NR	.37	5.0
**Pastor, priest, rabbi, reverend, or other church leader**						
Primary	5.7	4.8	9.9	5.9	.35	5.8
Specialty	7.2	3.3	9.9	NR	.007	6.8
**Spiritual healer**						
Primary	2.1	1.8	2.5	1.7	.90	1.9
Specialty	1.6	0	2.2	NR	.02	1.3
**Special diets**						
**Arthritis diet**						
Primary	7.8	8.3	9.9	2.0	<.001	5.9
Specialty	10.4	8.0	8.4	NR	.45	9.2
**Ayurvedic diet**						
Primary	0.5	0.3	0	0	.31	0.2
Specialty	0.2	0.3	0	NR	1.0	0.2
**Fasting/cleansing diet**						
Primary	4.7	2.3	6.2	1.0	.005	2.5
Specialty	1.4	1.3	6.2	NR	<.001	2.6
**Hypoglycemic diet**						
Primary	6.8	3.5	6.2	1.7	.01	3.6
Specialty	2.0	4.0	5.1	NR	.06	3.4
**Vegan diet**						
Primary	1.0	0.5	0	0.5	.84	0.6
Specialty	0.6	0.3	1.5	NR	.31	0.8
**Vegetarian diet**						
Primary	0	1.3	3.7	1.5	.08	1.3
Specialty	2.3	1.7	4.0	NR	.17	2.5
**Vitamins and minerals**						
**Beta carotene**						
Primary	7.8	5.8	11.1	5.0	.14	6.2
Specialty	5.1	5.3	6.9	NR	.56	5.6
**Copper**						
Primary	3.1	1.5	6.2	1.5	.03	2.1
Specialty	3.7	2.3	4.0	NR	.48	3.4
**Calcium**						
Primary	23.4	26.5	43.2	12.6	<.001	22.0
Specialty	42.9	29.0	33.9	NR	<.001	36.7
**Folic acid**						
Primary	7.8	6.8	13.6	6.7	.17	7.4
Specialty	37.4	8.0	12.0	NR	<.001	22.6
**Magnesium**						
Primary	7.3	7.8	23.5	5.9	<.001	8.2
Specialty	6.3	8.7	20.8	NR	<.001	10.7
**Niacin (vitamin B3)**						
Primary	3.7	4.0	7.4	4.5	.54	4.4
Specialty	4.3	4.7	7.7	NR	.12	5.3
**Pantothenic acid (vitamin B5)**						
Primary	2.6	2.8	7.4	2.5	.11	3.0
Specialty	3.1	3.7	5.8	NR	.16	4.0
**Selenium**						
Primary	2.1	2.0	8.6	2.7	.03	2.8
Specialty	3.3	4.0	6.6	NR	.09	4.3
**Vitamin B12**						
Primary	12.0	12.5	17.3	7.7	.03	11.0
Specialty	10.8	11.3	21.5	NR	<.001	13.7
**Vitamin C**						
Primary	17.2	15.5	22.2	9.4	.003	14.0
Specialty	20.7	17.7	23.7	NR	.20	20.6
**Vitamin D**						
Primary	11.5	12.8	17.3	6.7	.006	10.6
Specialty	17.4	14.3	16.4	NR	.53	16.3
**Vitamin E**						
Primary	22.9	19.0	30.9	11.6	<.001	17.8
Specialty	21.9	22.3	27	NR	.25	23.3
**Zinc**						
Primary	6.3	6.8	9.9	5.0	.37	6.2
Specialty	6.5	7.3	12.4	NR	.02	8.3
**Supplements**						
**Aloe vera**						
Primary	6.8	2.3	6.2	1.7	.002	3.2
Specialty	4.9	4.0	7.3	NR	.19	5.3
**Borage oil, black currant oil, or evening primrose oil**						
Primary	2.1	1.8	3.7	1.0	.26	1.7
Specialty	2.7	2.0	4.0	NR	.33	2.8
**Boron**						
Primary	0.5	0.5	3.7	0	.003	0.6
Specialty	0.2	0.7	1.5	NR	.08	0.7
**Baswellia, guggel**						
Primary	1.0	1.0	0	0.3	.50	0.7
Specialty	0.6	0.7	0.4	NR	>.99	0.6
**Bovine cartilage**						
Primary	0	0.8	0	0.7	.8	0.6
Specialty	1.0	1.0	0.7	NR	>.99	0.9
**Bromelain**						
Primary	0.5	2.0	2.5	0.3	.03	1.1
Specialty	1.0	1.0	1.5	NR	.87	1.1
**Cat's claw**						
Primary	1.0	1.0	2.5	0.7	.51	1.0
Specialty	0.8	1.0	1.8	NR	.50	1.1
**Cayenne**						
Primary	2.1	1.3	4.9	1.5	.15	1.8
Specialty	2.5	3.3	5.5	NR	.09	3.5
**Chondroitin**						
Primary	7.3	14.8	18.5	4.7	<.001	9.9
Specialty	10.0	23.0	17.2	NR	<.001	15.5
**Cod liver oil**						
Primary	3.7	3.3	1.2	2.5	.71	2.9
Specialty	5.1	4.3	4.7	NR	.88	4.8
**Copper**						
Primary	2.1	0.8	2.5	0.5	.11	1.0
Specialty	1.4	2.0	2.2	NR	.71	1.8
**Devil's claw**						
Primary	0	0.5	1.2	0	.12	0.3
Specialty	1.0	0.7	0.4	NR	.67	0.8
**Eucalyptus**						
Primary	2.1	0.5	2.5	1.0	.14	1.1
Specialty	1.2	0.7	0.7	NR	.78	0.9
**Fish oil**						
Primary	7.8	4.5	3.7	2.2	.02	4.2
Specialty	9.8	4.7	8.8	NR	.03	8.1
**Flaxseed oil**						
Primary	3.1	2.5	2.5	2.5	.96	2.6
Specialty	4.5	3.7	6.2	NR	.34	4.7
**Garlic**						
Primary	9.4	7.8	9.9	5.0	.14	7.2
Specialty	6.3	9.3	11.3	NR	.05	8.5
**Ginger**						
Primary	3.7	3.0	2.5	1.5	.30	2.5
Specialty	4.3	4.3	5.5	NR	.73	4.6
**Glucosamine**						
Primary	15.1	23.3	23.5	7.9	<.001	16.1
Specialty	18.0	31.0	25.2	NR	<.001	23.5
**Kava kava**						
Primary	1.0	1.3	2.5	1.0	.65	1.2
Specialty	1.0	0.7	5.8	NR	<.001	2.2
**Lei-gong-teng, Chinese tdundergod vine**						
Primary	0	0	0	0	>.99	0
Specialty	0.4	0	0.4	NR	.62	0.3
**Melatonin**						
Primary	1.0	1.3	8.6	1.2	.002	1.8
Specialty	1.6	2.0	10.6	NR	<.001	4.1
**Methylsulfonylmethane (MSM)**						
Primary	3.7	5.0	6.2	2.7	.28	4.0
Specialty	5.9	6.0	8.8	NR	.28	6.7
**Noni juice**						
Primary	1.0	0.3	1.2	0.5	.33	0.6
Specialty	1.2	1.0	1.1	NR	>.99	1.1
**S-adenosyl-L-methionine (SAM-e)**						
Primary	0	1.0	3.7	0.5	.04	0.8
Specialty	0.4	2.3	4.0	NR	.001	1.9
**Shark cartilage**						
Primary	2.1	2.5	9.9	1.7	.005	2.7
Specialty	5.3	1.7	4.7	NR	.04	4.1
**St. John's wort**						
Primary	3.1	4.0	7.4	3.0	.26	3.7
Specialty	2.0	2.7	9.5	NR	<.001	4.1
**Stinging nettle**						
Primary	0	0.3	0	0	.63	0.1
Specialty	0.4	0	0	NR	.50	0.2
**Turmeric**						
Primary	0.5	1.5	1.2	0.7	.57	1.0
Specialty	0.6	0.3	0.7	NR	.88	0.6
**Valerian root**						
Primary	2.1	1.3	6.2	1.2	.04	1.8
Specialty	0.6	1.0	8.8	NR	<.001	2.8
**Wild yam**						
Primary	1.0	0.8	1.2	1.2	.83	1.0
Specialty	0.4	0.7	0.7	NR	.77	0.6
**Ointments or topical rubs**						
**Arnica cream/gel**						
Primary	5.2	3.8	4.9	4.5	.81	4.4
Specialty	1.4	2.3	3.7	NR	.14	2.3
**Bengay, Icy Hot, or similar ointments or rubs**						
Primary	49.5	50.0	55.6	50.0	.81	50.3
Specialty	47.4	51.7	55.1	NR	.12	50.6
**Calendula**						
Primary	0.5	0.5	1.2	1.0	.74	0.7
Specialty	0.4	1.0	0.4	NR	.57	0.6
**Chamomile**						
Primary	2.1	1.0	6.2	2.5	.04	2.1
Specialty	0.8	3.0	3.3	NR	.02	2.1
**Clay**						
Primary	0	0.3	0	0.5	>.99	0.3
Specialty	0	0	0	NR	>.99	0
**Coriander cream**						
Primary	1.0	0.5	0	1.0	.81	0.7
Specialty	0.4	0.7	0.4	NR	.86	0.5
**Dimethyl sulfoxide (DMSO)**						
Primary	2.6	2.3	0	0.7	.14	1.6
Specialty	3.9	3.0	2.2	NR	.43	3.2
**Horse liniment**						
Primary	6.3	7.3	6.2	5.2	.70	6.2
Specialty	9.4	7.7	2.6	NR	.002	7.2
**Linseed oil**						
Primary	0.5	0.3	0	0.5	.88	0.4
Specialty	0.8	0.3	1.1	NR	.53	0.8
**MSM creams**						
Primary	1.0	0.5	2.5	1.0	.32	0.9
Specialty	2.0	1.3	2.2	NR	.71	1.9
**Pine tree sap**						
Primary	1.0	0.3	1.2	0.3	.25	0.5
Specialty	0.8	0.3	0	NR	.39	0.5
**Rosemary**						
Primary	2.1	0.3	2.5	1.5	.05	1.2
Specialty	0.8	0.7	0.4	NR	.89	0.7
**Sesame oil**						
Primary	0.5	0.3	1.2	0.3	.36	0.4
Specialty	0.6	0	1.1	NR	.18	0.6
**Tiger balm, white flower oil**						
Primary	4.2	3.5	3.7	2.7	.76	3.3
Specialty	4.1	4.3	6.9	NR	.19	4.9
**Traumeel or traumed ointment**						
Primary	0.5	0.3	0	0.5	.88	0.4
Specialty	0.2	0.3	0.4	NR	>.99	0.3
**Volcanico**						
Primary	0	0.3	0	0	.63	0.1
Specialty	0.2	0.7	0	NR	.46	0.3
**Body treatments**						
**Acupressure beads/seeds**						
Primary	1.0	1.8	6.2	1.2	.04	1.8
Specialty	0.6	1.7	4.0	NR	.004	1.8
**Copper bracelet or copper jewelry**						
Primary	20.8	18.0	18.5	11.1	.007	16.0
Specialty	29.7	23.0	21.9	NR	.03	25.8
**Herbal plasters**						
Primary	0	1.0	1.2	0	.05	0.5
Specialty	0.2	0.7	1.1	NR	.25	0.6
**Infrared wraps**						
Primary	1.0	1.0	2.5	0	.03	0.7
Specialty	1.6	0.7	2.6	NR	.20	1.6
**Magnets**						
Primary	10.9	12.3	19.8	9.4	.06	11.5
Specialty	15.5	18.0	28.5	NR	<.001	19.6
**Q-Ray bracelet (ionically charged bracelet)**						
Primary	1.6	0.5	0	0.5	.47	0.7
Specialty	0.8	0.3	1.8	NR	.20	0.9
**Movement**						
**Alexander movement technique**						
Primary	0	1.8	1.2	1.0	.26	1.1
Specialty	0.6	0	0.4	NR	.39	0.4
**Feldenkrais method (awareness through movement)**						
Primary	0	1.8	0	1.0	.23	1.0
Specialty	1.0	1.0	1.8	NR	.56	1.2
**Pilates movements**						
Primary	0.5	1.0	0	0.7	.95	0.7
Specialty	1.0	1.3	2.6	NR	.25	1.5
**Qi gong (chi kong)**						
Primary	0	0.8	3.7	0.5	.04	0.7
Specialty	0.4	0.7	1.8	NR	.14	0.9
**Tai chi**						
Primary	2.1	2.3	2.5	1.2	.63	1.9
Specialty	0.8	2.7	4.7	NR	.002	2.4
**Trager approach (Mentastics)**						
Primary	0	0.5	0	0	.58	0.2
Specialty	0.2	0.3	1.1	NR	.17	0.5
**Yoga**						
Primary	3.1	4.5	12.4	3.7	.005	4.6
Specialty	3.9	5.3	14.2	NR	<.001	7.0
**Spiritual**						
**Attend religious services regularly**						
Primary	19.3	16.8	32.1	14.6	.002	17.6
Specialty	23.5	19.0	24.1	NR	.25	22.4
**Draw on religious or spiritual beliefs**						
Primary	32.8	26.0	55.6	18.3	<.001	26.6
Specialty	32.7	27.7	48.2	NR	<.001	35.3
**Pray about your arthritis**						
Primary	14.1	13.5	27.2	10.9	.002	13.7
Specialty	14.7	15.0	24.8	NR	.001	17.4
**Mind-body therapies**						
**Meditate**						
Primary	49.0	35.8	55.6	26.2	<.001	36.0
Specialty	47.4	34.7	52.9	NR	<.001	45.3
**Relax each muscle group or part of the body one after another**						
Primary	16.7	16.0	43.2	14.9	<.001	17.7
Specialty	15.1	15.3	33.9	NR	<.001	20.0
**Sing, make sounds, or play, or use a musical instrument**						
Primary	8.9	10.3	27.2	8.9	<.001	10.8
Specialty	14.3	13.3	25.2	NR	<.001	16.8
**Use special breathing techniques**						
Primary	7.8	10.0	34.6	7.4	<.001	10.5
Specialty	11.3	12.3	29.2	NR	<.001	16.2
**Visualization**						
Primary	8.3	6.0	30.9	4.2	<.001	7.6
Specialty	8.0	7.3	22.6	NR	<.001	11.6

Abbreviations: CAM, complementary and alternative medicine; NR, not reported.

a *P* value within North Carolina Family Medicine Research Network or specialist χ^2^.

b A Mexican practitioner of traditional Mayan healing techniques.
